# MUSCLE UP: WHAT AGING LOOKS LIKE FROM THE INSIDE OUT

**DOI:** 10.1093/geroni/igad104.0583

**Published:** 2023-12-21

**Authors:** Eleanor Simonsick, Luigi Ferrucci

## Abstract

The Longitudinal Studies Section of the Translational Gerontology Branch of the National Institute on Aging Intramural Research Program has the mission and opportunity to examine aging physiology from the cellular to the full body functional level in model systems and human populations. This symposium presents ongoing research that targets pathologic and normative change in muscle with aging and the significance of these changes for understanding the aging process. Data come from the Baltimore Longitudinal Study of Aging (BLSA) and the Genetic and Epigenetic Signatures of Translational Aging Laboratory Testing (GESTALT) study. In nearly 500 BLSA participants aged 50+, Zenobia Moore evaluates the association between multiple epigenetic age metrics and change over 6 years in mid-femur areas of muscle and subcutaneous and intramuscular fat derived from computed tomography. Qu (Teresa) Tian investigates the relationship between skeletal muscle mitochondrial function and risk of MCI or dementia over 3.3 years in 460 initially cognitively normal BLSA participants and PET and blood biomarkers of AD in available subsamples. In 82 men and women from GESTALT, Stefano Donega examines relationships between physical activity, peak oxygen consumption and muscle oxidative capacity using p31 MR spectroscopy and gene expression, transcript usage and splicing events derived from vastus lateralis biopsies. Using volumetric Focused Ion Beam Scanning Electron Microscopy, Lisa Hartnell shares 3D images of muscle samples at the level of the mitochondrial reticulum and sarcomere from young and old healthy and frail GESTALT participants. Together, these findings highlight the value of muscle as a window on aging.

